# Heparin Induced Thrombocytopenia: Case Presentation and Review

**DOI:** 10.4021/jocmr751w

**Published:** 2012-01-17

**Authors:** Ronny A. Cohen, Mariely Castellano, Christine A. Garcia

**Affiliations:** a Woodhull Medical Center, NYU School of Medicine, Brooklyn, NY, USA; b St. George’s University School of Medicine, Grenada

## Abstract

Heparin Induced Thrombocytopenia (HIT) is an adverse effect of heparin therapy with possibly devastating consequences. We present a case of HIT Type II. An in-depth review of HIT is presented, examining the important clinical symptoms and diagnostic indicators. The treatment of HIT is then discussed, with an emphasis on current therapies. An extensive literature review has been performed to present a comprehensive review of the causes, pathophysiology and treatment of HIT.

**Keywords:**

Heparin induced thrombocytopenia; Anticoagulation; PF4; HIT

## Introduction

Heparin Induced Thrombocytopenia (HIT) is defined as a prothrombotic adverse effect of heparin therapy [1]. There are two types of HIT described. Type I is a non-immune, mediated, asymptomatic, transient drop in platelet count that occurs in some heparin treated patients. It is typically characterized by a lesser fall in platelet count within the first two days after heparin initiation and often returns to normal with continued heparin administration [2]. The more serious form is Type II (HIT-II) is an immune-mediated disorder characterized by the formation of antibodies against heparin-platelet factor 4 complexes. Since HIT Type I is of no clinical significance, this paper discusses HIT Type II. The frequency of HIT varies from 0.5% to 5%, depending on the patient population studied [3]. A meta-analysis noted an incidence of 2.6 percent [4].

## Case History

A 70 years old woman was recently discharged following exploratory laparotomy for perforated duodenal ulcer with peritonitis. The patient presented on discharge day 4 with complaints of right-sided pleuritic chest pain that started the day after discharge. Pain was described as dull in nature, 5/10 in severity, radiating to the right upper quadrant, aggravated by deep respiration and associated with cough productive of whitish sputum. The patient admitted to having chills but denies fever, shortness of breath or palpitations. The patient denied diarrhea or constipation. The patient denied having bloody or black stools. Her medical history included hypothyroidism, hypertension, GERD and peptic ulcer disease.

Physical examination of the patient revealed an obese woman in mild distress. Her blood pressure was 122/77 mmHg with a pulse of 86 beats per minute. She was breathing 16 times/minute with an oxygen saturation of 100%. She was afebrile. On head and neck examination, her pupils were equal, round, reactive to light and accommodation. Her sclera was anicteric, revealing mild pallor. On cardiovascular exam, heart sounds were audible with regular rate and rhythm, normal S1 and S2, no murmurs. Her lung fields had decreased air entry bilaterally, right side greater than left. Her abdomen had surgical stables present along the midline with tenderness around the surgical site. Her extremities were symmetric without tenderness, cyanosis or pedal edema. Her pulses were present and palpable bilaterally.

Her blood chemistry panel revealed a serum sodium of 132 mmol/L, potassium of 3.6 mmol/L, chloride of 102 mmol/L, bicarbonate of 22 mmol/L, BUN of 15 mg/dL, creatinine of 1.0 mg/dL and glucose of 92 mg/dL. Her Leukocyte count was 16.0 x10^3^ per µL, with 83% neutrophils. Her hemoglobin was 10.1 g/dL, hematocrit 29.5% and platelet count was 170 x10^3^ per µL. Arterial blood gas sampling showed a pH of 7.48, PaCO2 of 33 mmHg, PaO2 of 77 mmHg, and 97% and FiO2 of 21%. Chest x-ray revealed pneumonia in the right lung. The patient was diagnosed with hospital-acquired pneumonia and treated with intravenous fluids, IV Cefepime and Ciprofloxacin, Levothryoxine, Nexium and Lotrel.

On hospital day 2, the patient reported improvement of symptoms. Vital signs were within normal limits. Leukocyte count was 8.0 x10^3^ per µL. Hemoglobin was 8.6 g/dL, hematocrit was 26% and platelet count was 118 x10^3^ per µL. CT scan revealed an improving pleural effusion. CBC was monitored with a discharge plan if leukocytes were trending downward. In the evening, the patient complained of left knee pain. Physical exam revealed erythema around the lateral aspect of the left knee. Patient denied any trauma but states that the Flow-tron was a little tight. The Flow-tron was loosened and Tylenol was given for pain management. An hour later, the patient was re-evaluated. The entire left leg was noted to be swollen and now tender to touch. An assessment of deep vein thrombosis (DVT) was made and the patient was started on heparin infusion.

On hospital day 5, lower extremity duplex scan showed acute thrombosis of left common femoral, superficial femoral, popliteal, tibial and saphenous veins, with absence of flow and compressibility. The right popliteal vein also showed chronic re-canalized thrombosis. Leukocyte count was 9.9 x10^3^ per µL, hemoglobin was 8.5 g/dL. Hematocrit 24.7%, platelet count was 89.2 x10^3^ per µL. Platelets on admission were 170 x10^3^ per µL. Serotonin release assay was 100%. The patient was diagnosed with HIT and started on Lepirudin. The leg swelling improved and platelet count improved to 197 x10^3^ per µL.

## Discussion

### Clinical presentation

The initial sign of HIT usually is the development of thrombocytopenia. The major manifestations of venous thrombosis are deep vein thrombosis (DVT) and pulmonary embolism (PE). Other manifestations include venous limb gangrene and cerebral sinus thrombosis [5]. Upper extremity DVT has also been described in HIT, but it is less common than lower extremity DVT [6]. Most upper extremity DVT occurred in patients with central venous catheters (CVC) and at the CVC site. Arterial thrombosis is less common in HIT but can lead to significant clinical complications including stroke, myocardial infarction, acute limb ischemia from peripheral arterial occlusion, or kidney infarction. White clot syndrome syndrome refers to platelet-rich aggregations leading to arterial thrombosis [7]. Finally, skin necrosis on fat-rich areas such as the abdomen are commonly seen in HIT. Similar to warfarin-induced skin necrosis, erythema is followed by purpura and hemorrhage leading to necrosis, however there are no deficiencies in anticoagulants [8].

### Onset

There are three patterns for the onset of thrombocytopenia related to heparin exposure described [1,9]. The most common pattern, typical-onset of HIT, occurs in 4 - 10 days after initial exposure to heparin with a fall in platelet count of > 50 percent. The second pattern, delayed onset HIT, occurs a mean of 9 days after heparin stopped, but can be as long as 40 days [10-12]. Delayed HIT may account for 13 - 15% of all cases of HIT [13]. Early onset of HIT may be seen in about 30% of patients with persistent antibodies due to heparin therapy within the previous one to three months. The median time of platelet fall was 10.5 hours after the start of heparin administration [14].

### Risk factors

The risk of developing HIT is related to many factors, including the type of heparin product administered, route of administration, duration of therapy, patient population and previous exposure to heparin [15]. The use of unfractionated heparin (UFH) rather than low molecular weight heparin (LMWH), surgical rather than medical patients and female sex, have been associated with greater risk of developing HIT. Some surgical populations, such as cardiac transplant or neurosurgery patients have somewhat higher risk [16,17]. Antibodies are more likely to form in patients undergoing cardiac surgery with an incidence as high as 15 to 20% than in orthopedic patients. Interestingly, orthopedic surgery patients compared with cardiac surgery patients, who are receiving unfractionated heparin post-operatively are less likely to develop heparin PF4-antibodies (14% versus 50% by antigen assay), yet more likely to develop HIT (4.9% versus 1.0%) [18,19]. HIT was rarely found among patients less than 40 years of age as well as in women following delivery [20].

### Pathophysiology

Following administration of heparin, a heparin neutralizing protein present within platelets (PF4) binds to heparin. This complex provokes the formation of antibodies, IgG and IgM, which bind to the heparin-PF4 complex. This leads to platelet activation. Platelet activation leads to release of more PF4 with subsequent positive feedback. Also, platelet activation leads to platelet aggregation and premature removal which results in thrombocytopenia, release of micro-particles with pro-thrombic activity. Microvascular endothelial cells are activated, resulting in the release of interleukin-6, von Willebrand factor, and other adhesion molecules.

### Diagnosis

Since time of onset can range between hours to several weeks, clinical suspicion for HIT is important in recognizing and starting therapy. The diagnosis of HIT is made on clinical grounds since assays generally take a long time, whereas immediate therapy is critical to avoid often fatal complications of HIT. The following clinical scenarios should raise clinical suspicion in any patient received LMWH: onset of otherwise unexplained thrombocytopenia, venous or arterial thrombosis associated with thrombocytopenia, a platelet count that has fallen 50 percent or more from a precious value, necrotic skin lesions at heparin injection sites or acute systemic reactions subsequent to IV heparin bolus administration [21].

The criteria used in the diagnosis of HIT include [22]: (1) Normal platelet count before commencement of heparin. (2) Thrombocytopenia defined as a drop in platelet by 30% or a drop of 50% from patient baseline. (3) Onset of thrombocytopenia typically 5 - 10 days after initiation of heparin treatment. (4) Acute thrombotic event. (5) Exclusion of thrombocytopenia after cessation of heparin. (6) HIT antibody seroconversion.

Diagnosis can be made utilizing a clinical scoring system based on the pretest probability of HIT known as the 4Ts: Thrombocytopenia, Thrombosis, Timing, and other causes [23]. A score is determined based on the scoring system summarized in Table 1. A score from 0 - 3 denotes low probability, 4 - 5 denotes an intermediate probability and 6 - 8 denotes a high probability of HIT.

**Table 1:** Pretest Probability of HIT

**Pretest probability of HIT**	**Score**
Thrombocytopenia	
Platelet count fall > 50% and nadir > 20,000	2 points
Platelet count fall 30 - 50 % or nadir 10 to 19,000	1 point
Platelet count fall < 30% or nadir < 10,000	0 points
Timing of platelet count fall	
Clear onset between days 5 and 10 or platelet count fall at ≤ 1 day if prior heparin exposure within the last 30 days	2 points
Consistent with fall at 5 to 10 days but unclear (eg, missing platelet counts), onset after day 10, or fall ≤ 1 day with prior heparin exposure within 30 to 100 days	1 point
Platelet count fall at < 4 days without recent exposure	0 points
Thrombosis	
Confirmed new thrombosis, skin necrosis, or acute systemic reaction after IV unfractionated heparin bolus	2 points
Progressive or recurrent thrombosis, non-necrotizing (erythematous) skin lesions, or suspected thrombosis which has not been proven	1 point
None	0 points
Other causes	
None apparent	2 points
Possible	1 point
Definite	0 points

### Diagnostic tests

Two general types of assays can be used to detect antibodies [24,25]. Most widely commercial enzyme immunoassays test for antibodies reactive against PF4/heparin or PF4/polyvinyl sulfonate. ELISA test are very sensitive (91% to > 97%), whereas a negative test strongly suggests the absence of HIT. In contrast, platelet activation assays detect HIT antibodies upon their platelet-activating properties. The gold standard for diagnostic tests for HIT is the 14 C-serotonin release assay. A positive test is the release of 14 C-seroton when therapeutic (0.1 U/ML) concentrations are used, rather than high (100 U/mL) concentrations. A positive test was stongly associated with thrombocytopenia starting 5 or more days after heparin exposure [26]. Heparin-induced platelet aggregation assay is a specific test for diagnosing HIT, but lacks sensitivity [25]. A positive test shows low background aggregation with no added heparin, aggregation with the addition of a low concentration of heparin, and absent aggregation with high heparin concentration.

### Current therapies

When the diagnosis of HIT is confirmed, therapeutic doses of alternative non-heparin anticoagulants are usually required. Heparin treatments must be stopped immediately including heparin-bonded catheters and heparin flushes. Patients remain at risk for thrombosis from heparin cessation alone. Patients should be given a non-heparin anticoagulant. Coumadin should not be given. Prophylactic platelet transfusions should not be given [11].

### Direct thrombin inhibitors

Direct thrombin inhbitors which include Bivalirudin, Argatroban and Lepiridin, directly inhibit procoagulant and prothrombotic actions of thrombin. They do not require a cofactor to inhibit thrombin. They are active against both free and clot-bound thrombin. They do not interact with or produce heparin dependent antibodies.

## References

[1] Warkentin TE (2003). Heparin-induced thrombocytopenia: pathogenesis and management. Br J Haematol.

[2] Warkentin TE, Greinacher A, Koster A, Lincoff AM (2008). Treatment and prevention of heparin-induced thrombocytopenia: American College of Chest Physicians Evidence-Based Clinical Practice Guidelines (8th Edition). Chest.

[3] Jang IK, Hursting MJ (2005). When heparins promote thrombosis: review of heparin-induced thrombocytopenia. Circulation.

[4] Martel N, Lee J, Wells PS (2005). Risk for heparin-induced thrombocytopenia with unfractionated and low-molecular-weight heparin thromboprophylaxis: a meta-analysis. Blood.

[5] Warkentin TE, Kelton JG (1996). A 14-year study of heparin-induced thrombocytopenia. Am J Med.

[6] Warkentin TE, Elavathil LJ, Hayward CP, Johnston MA, Russett JI, Kelton JG (1997). The pathogenesis of venous limb gangrene associated with heparin-induced thrombocytopenia. Ann Intern Med.

[7] Stanton PE, Jr (1988). , Evans JR, Lefemine AA, Vo NM, Rannick GA, Morgan CV, Jr., Hinton PJ, et al. White clot syndrome. South Med J.

[8] Sallah S, Thomas DP, Roberts HR (1997). Warfarin and heparin-induced skin necrosis and the purple toe syndrome: infrequent complications of anticoagulant treatment. Thromb Haemost.

[9] Warkentin TE, Kelton JG (2001). Temporal aspects of heparin-induced thrombocytopenia. N Engl J Med.

[10] Rice L, Attisha WK, Drexler A, Francis JL (2002). Delayed-onset heparin-induced thrombocytopenia. Ann Intern Med.

[11] Warkentin TE, Kelton JG (2001). Delayed-onset heparin-induced thrombocytopenia and thrombosis. Ann Intern Med.

[12] Smythe MA, Stephens JL, Mattson JC (2005). Delayed-onset heparin-induced thrombocytopenia. Ann Emerg Med.

[13] Frame JN, Davis E, Reed J, Wang Y, Emmett JS (2004). Clinical features and outcomes in patients with heparin-induced thrombocytopenia (HIT): report of the CAMC registry (abstract). Blood.

[14] Warkentin TE, Makris M, Jay RM, Kelton JG (2008). A spontaneous prothrombotic disorder resembling heparin-induced thrombocytopenia. Am J Med.

[15] Dager WE, Dougherty JA, Nguyen PH, Militello MA, Smythe MA (2007). Heparin-induced thrombocytopenia: treatment options and special considerations. Pharmacotherapy.

[16] Hourigan LA, Walters DL, Keck SA, Dec GW (2002). Heparin-induced thrombocytopenia: a common complication in cardiac transplant recipients. J Heart Lung Transplant.

[17] Hoh BL, Aghi M, Pryor JC, Ogilvy CS (2005). Heparin-induced thrombocytopenia Type II in subarachnoid hemorrhage patients: incidence and complications. Neurosurgery.

[18] Warkentin TE, Sheppard JA, Horsewood P, Simpson PJ, Moore JC, Kelton JG (2000). Impact of the patient population on the risk for heparin-induced thrombocytopenia. Blood.

[19] Bauer TL, Arepally G, Konkle BA, Mestichelli B, Shapiro SS, Cines DB, Poncz M (1997). Prevalence of heparin-associated antibodies without thrombosis in patients undergoing cardiopulmonary bypass surgery. Circulation.

[20] Stein PD, Hull RD, Matta F, Yaekoub AY, Liang J (2009). Incidence of thrombocytopenia in hospitalized patients with venous thromboembolism. Am J Med.

[21] Warkentin TE, Hirte HW, Anderson DR, Wilson WE, O'Connell GJ, Lo RC (1994). Transient global amnesia associated with acute heparin-induced thrombocytopenia. Am J Med.

[22] Warkentin TE, Aird WC, Rand JH Platelet-endothelial interactions: sepsis, HIT, and antiphospholipid syndrome. Hematology Am Soc Hematol Educ Program.

[23] Linkins LA, Warkentin TE (2008). The approach to heparin-induced thrombocytopenia. Semin Respir Crit Care Med.

[24] Warkentin TE, Greinacher A, Warkentin TE, Greinacher A (2001). Laboratory testing for heparin-induced thrombocytopenia. Heparin-Induced Thrombocytopenia, 4th ed.

[25] Warkentin TE (2002). Platelet count monitoring and laboratory testing for heparin-induced thrombocytopenia. Arch Pathol Lab Med.

[26] Napolitano LM, Warkentin TE, Almahameed A, Nasraway SA (2006). Heparin-induced thrombocytopenia in the critical care setting: diagnosis and management. Crit Care Med.

